# Repositioning of a cyclin-dependent kinase inhibitor GW8510 as a ribonucleotide reductase M2 inhibitor to treat human colorectal cancer

**DOI:** 10.1038/cddiscovery.2016.27

**Published:** 2016-05-09

**Authors:** Y-Y Hsieh, C-J Chou, H-L Lo, P-M Yang

**Affiliations:** 1 PhD Program for Cancer Biology and Drug Discovery, College of Medical Science and Technology, Taipei Medical University and Academia Sinica, Taipei, Taiwan; 2 Division of Hematology and Oncology, Shuang Ho Hospital, Taipei Meidcal University, Taipei, Taiwan; 3 Graduate Institute of Cancer Biology and Drug Discovery, College of Medical Science and Technology, Taipei Medical University, Taipei, Taiwan

## Abstract

Colorectal cancer (CRC) is the second leading cause of cancer-related death in males and females in the world. It is of immediate importance to develop novel therapeutics. Human ribonucleotide reductase (RRM1/RRM2) has an essential role in converting ribonucleoside diphosphate to 2′-deoxyribonucleoside diphosphate to maintain the homeostasis of nucleotide pools. RRM2 is a prognostic biomarker and predicts poor survival of CRC. In addition, increased RRM2 activity is associated with malignant transformation and tumor cell growth. Bioinformatics analyses show that RRM2 was overexpressed in CRC and might be an attractive target for treating CRC. Therefore, we attempted to search novel RRM2 inhibitors by using a gene expression signature-based approach, connectivity MAP (CMAP). The result predicted GW8510, a cyclin-dependent kinase inhibitor, as a potential RRM2 inhibitor. Western blot analysis indicated that GW8510 inhibited RRM2 expression through promoting its proteasomal degradation. In addition, GW8510 induced autophagic cell death. In addition, the sensitivities of CRC cells to GW8510 were associated with the levels of RRM2 and endogenous autophagic flux. Taken together, our study indicates that GW8510 could be a potential anti-CRC agent through targeting RRM2.

Colorectal cancer (CRC), one of the most common cancers worldwide, is the second leading cause of cancer-related death in males and females in the world. Despite advances in surgical techniques, adjuvant therapy, and molecular targeted therapy, there is only a modest increase in cancer patient survival.^1^ Thus, developing novel therapeutic strategies is of immediate importance. Human ribonucleotide reductase (RR) is a heterotetramer consisting of two large RRM1 subunits and two small RRM2 subunits. RR has an essential role in converting ribonucleoside diphosphate to 2′-deoxyribonucleoside diphosphate to maintain the homeostasis of nucleotide pools.^2^ The critical role played by RR in DNA synthesis and repair has identified it as an attractive target for anticancer agents.^3^ In addition, increased RR activity is associated with malignant transformation and tumor cell growth,^4^ suggesting that inhibition of RR might have the potential to treat cancers.

Drug repositioning or repurposing refers to find new indications of clinically used drugs or compounds that failed during development.^5^ The advantages of drug repositioning are the reduction of costs and the bypass of safety concerns.^5^ However, it is still challenging to discover new indications with drug repositioning. More and more biomedical databases have been developed in recent years, and utilization of these resources would be highly useful for drug repositioning.^6^ For example, the connectivity MAP (CMAP) database collects gene expression profiles from small molecule-treated human cancer cells. The current version (build 02) of CMAP contains more than 7000 expression profiles representing 1309 compounds. By comparing gene expression signatures, this tool provides connections among small molecules sharing a mechanism of action, chemicals and physiological processes, and diseases and drugs.^7^ Because most of CMAP compounds are FDA-approved drugs, CMAP becomes a powerful tool for drug repositioning.

In this study, we found that RRM2 might be a potential molecular target for treating CRC. We mined the CMAP database to discover novel RRM2 inhibitors. We identified that GW8510, a cyclin-dependent kinase, inhibited RRM2 expression through promoting its degradation. In addition, GW8510 induced autophagic cell death in human CRC cells. The sensitivities of CRC cells were correlated with the endogenous RRM2 level and intrinsic autophagic flux. Our results reposition GW8510 as a novel RRM2 inhibitor for treating CRC.

## Results

### RRM2 is a therapeutic target for treating CRC

**Figure 1:**
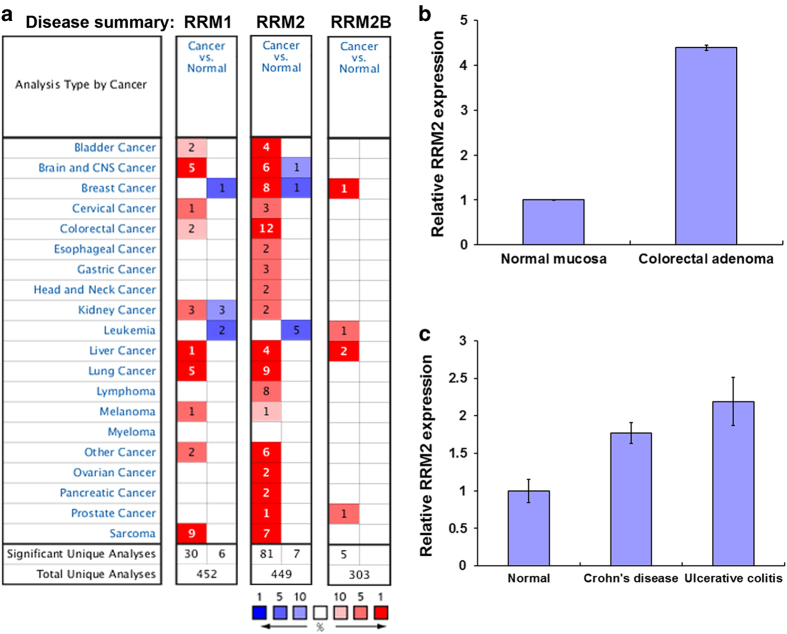
Role of RRM2 in CRC. (**a**) Summary view of RRM1, RRM2, and RRM2B expression profiles in human tumors using published human oncology microarray data (Oncomine). The number in each cell under ‘Cancer *versus* Normal’ corresponds to the amount of cancer types that contains a significantly different level of RRM1, RRM2, or RRM2B compared with normal corresponding tissue. Thresholds for significance are: fold expression>2; *P*-value<0.05 and ranking of gene in the analyses>top 10%. Red signifies the gene overexpression in the analyses; blue represents the gene underexpression. Intensity of color signifies the best rank of gene in those analyses. (**b**) A microarray dataset (GSE8671) of colorectal adenomas and adjacent normal mucosa was obtained from NCBI GEO database. Probe IDs of RRM2: 209773_s_at. (**c**) A microarray dataset (GSE1710) of patients with Crohn’s disease and ulcerative colitis was obtained from NCBI GEO database. RRM2 expression in these datasets was shown.

RR consists of two protein subunits, RRM1 and RRM2. In addition, RRM2 can be substituted by a p53-inducible small subunit RRM2B (p53R2) that is involved in the synthesis of dNTPs required for DNA damage repair.^8^ To investigate the roles of RR subunits in CRC, a pan-cancer analysis for the expressions of RRM1, RRM2, and RRM2B in normal and cancerous tissues was performed by using a complete collection of human cancer microarray data (Oncomine database).^9^ As shown in Figure 1a, RRM2 was frequently overexpressed in various cancer datasets (81 out of 449 analyses), except for leukemia, compared with RRM1 (30 out of 452 analyses) and RRM2B (5 out of 303 analyses). In addition, 12 out of 81 RRM2-overexpressing datasets belong to CRC analyses. Therefore, RRM2 was frequently overexpressed in CRC.

**Figure 2:**
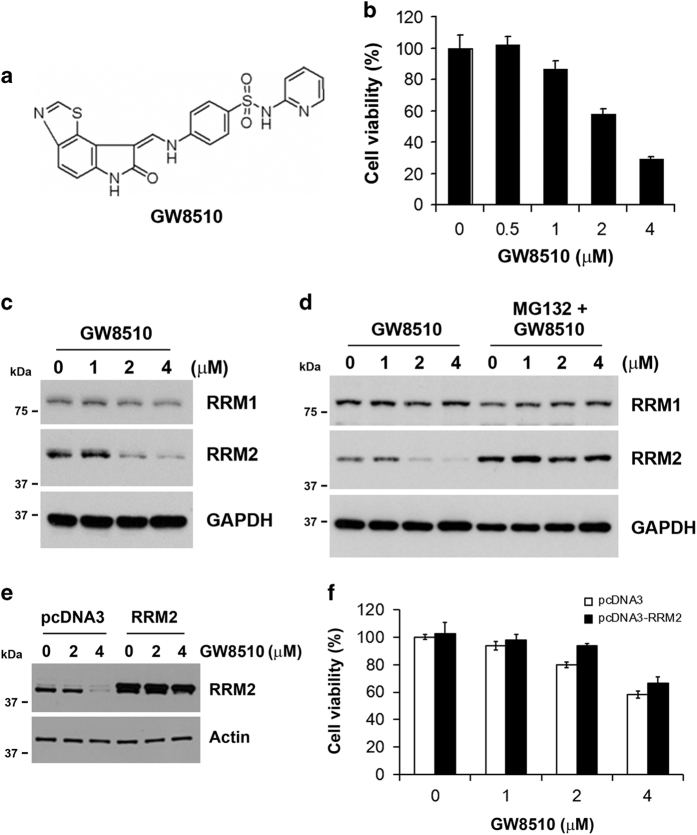
Identification of GW8510 as a potential RRM2 inhibitor. (**a**) The chemical structures of GW8510. (**b**) HCT116 cells were treated with various doses of GW8510 for 72 h. The cell viability was analyzed by an MTT assay. (**c**) HCT116 cells were treated with various doses of GW8510 for 24 h. The protein expressions were analyzed by western blots. (**d**) HCT116 cells were treated with various doses of GW8510 for 24 h in the absence or presence of 5 *μ*M MG132. The protein expressions were analyzed by western blots. (**e**) HCT116 cells were transiently transfected with a RRM2-overexpressing (pcDNA3-RRM2) or a control (pcDNA3) plasmid for 48 h, and then treated with indicated doses of GW8510 for 24 h. The protein expressions were analyzed by western blots. (**f**) HCT116 cells were transiently transfected with a RRM2-overexpressing (pcDNA3-RRM2) or a control (pcDNA3) plasmid for 24 h, and then treated with indicated doses of GW8510 for 72 h. The cell viability was analyzed by an MTT assay.

To further investigate the essential role of RRM2 in CRC, its expression in colorectal adenomas and adjacent normal mucosa of CRC patients was analyzed from a published microarray dataset (GSE8671^10^) obtained from the NCBI Gene Expression Omnibus (GEO) database.^11^ Adenomatous polyps are believed to be the precancerous lesions of CRC.^12^ As shown in Figure 1b, the level of RRM2 was higher in adenomas. Inflammatory bowel disease, including Crohn’s disease and ulcerative colitis, is an important risk factor for the development of colitis-associated CRC.^13,14^ The expression of RRM2 was also higher in patients with Crohn’s disease and ulcerative colitis (dataset: GSE1710^15^) (Figure 2b). These results suggest that RRM2 may promote CRC tumorigenesis and may be an attractive therapeutic target for treating CRC.

### Identification of GW8510 as a potent RRM2 inhibitor for treating CRC

To develop novel RRM2 inhibitors, the CMAP database was used to search possible inhibitors of RRM2. Differentially expressed genes (Supplementary Table S1) were prepared from microarray data (GSE15212) of RRM2-knockdown SW480 human CRC cells.^16^ This gene set was queried by CMAP and the result was listed in Table 1 according to the given rank. The CMAP drugs with positive mean scores have similar gene expression profiles when RRM2 was knocked down in cells. The rank 1 compound is phenoxybenzamine, a non-selective, irreversible alpha antagonist. The major type of drugs belong to cyclin-dependent kinase inhibitors (GW8510 and 0175029-0000) and topoisomerase II inhibitors (doxorubicin, daunorubicin and ellipticine). Because the discovery of these compounds was based on their similarity to the gene expression profile of RRM2-knockdown cells, we propose that these compounds may reduce the expression of RRM2. To demonstrate this possibility, human CRC cells, HCT116 or DLD-1, were treated with phenoxybenzamine (Supplementary Figure S1A), doxorubicin, daunorubicin (Supplementary Figure S2A), and GW8510 (Figure 2A) for 24 h, and then cell viability assay and western blot analysis were performed. Phenoxybenzamine did not alter cell viability and the protein expression of RRM1 and RRM2 (Supplementary Figures S1B and S1C). Both doxorubicin and daunorubicin inhibited cell viability but induced RRM2 expression (Supplementary Figures S2B and S2C). In contrast, GW8510 inhibited both cell viability and RRM2 expression without alteration of RRM1 expression (Figures 2b and c). The reduction of RRM2 protein level by GW8510 can be reversed by MG132, a proteasome inhibitor (Figure 2d), suggesting that GW8510 promotes the degradation of RRM2 protein. To study whether the reduction of cell viability by GW8510 was dependent on RRM2 inhibition, a RRM2-overexpression plasmid was transfected into HCT116 cells (Figure 2e), and then exposed to GW8510. The cell viability was examined by a MTT assay. As shown in Figure 2f, overexpression of RRM2 rescued cells from the growth-inhibitory effect of GW8510. Therefore, GW8510 could inhibit cell viability of HCT116 cells through targeting the RRM2 protein and promoting its degradation.

### GW8510 induces autophagic cell death of CRC cells

**Figure 3:**
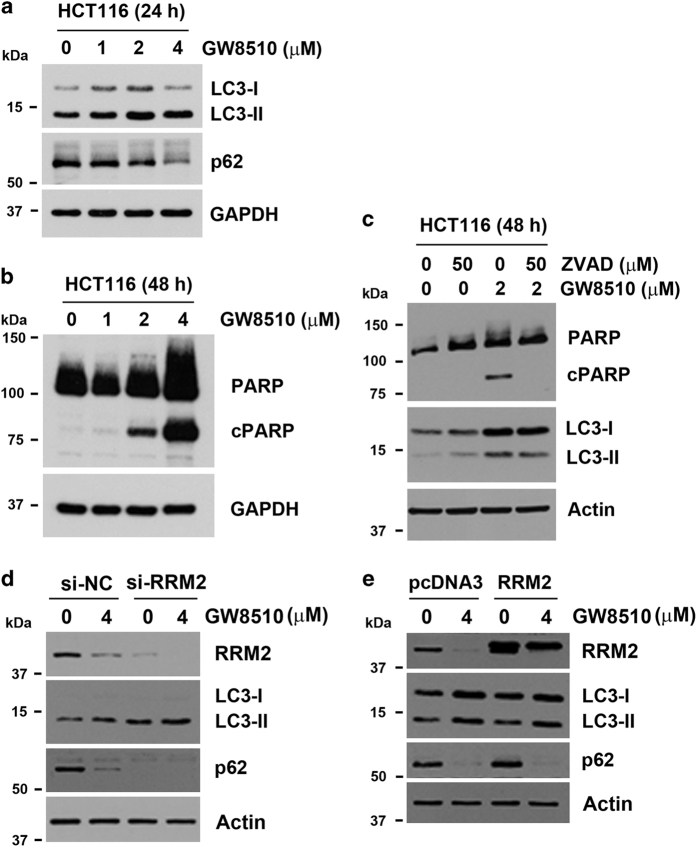
GW8510 induced autophagic cell death of CRC cells. (**a**) HCT116 cells were treated with various doses of GW8510 for 24 h. The protein expressions were analyzed by western blots. (**b**) HCT116 cells were treated with various doses of GW8510 for 48 h. The protein expressions were analyzed by western blots. (**c**) HCT116 cells were treated with 2 *μ*M GW8510 for 48 h in the absence or presence of 50 *μ*M ZVAD-FMK. The protein expressions were analyzed by western blots. (**d**) HCT116 cells were transiently transfected with RRM2 siRNA for 48 h, and then treated with 4 *μ*M GW8510 for 24 h. The protein expressions were analyzed by western blots. (**e**) HCT116 cells were transiently transfected with a RRM2-overexpressing (pcDNA3-RRM2) or a control (pcDNA3) plasmid for 48 h, and then treated with 4 *μ*M GW8510 for 24 h. The protein expressions were analyzed by western blots.

To investigate the involved mechanism for the anti-CRC effect of GW8510, cell apoptosis was examined by the cleavage of PARP. As shown in Figure 3a, GW8510 induced the cleavage of PARP in a dose-dependent manner. Autophagy (autophagic cell death) is an alternative form of programmed cell death.^17^ Recently, inhibition of RRM2 has been demonstrated to trigger autophagy.^18^ To investigate whether GW8510 also induced autophagy, HCT116 cells were treated with GW8510, and then two autophagy markers, LC3-II and p62,^19^ were examined by western blot analysis. As shown in Figure 3b, GW8510 induced the accumulation of LC3-II and the downregulation of p62, suggesting the induction of autophagy by GW8510. To investigate the interplay between apoptosis and autophagy, a caspase-3 inhibitor, ZVAD-FMK, was used to inhibit apoptosis. As shown in Figure 3c, ZVAD-FMK inhibited GW8510-induced PARP cleavage. Interestingly, ZVAD-FMK also inhibited LC3-II accumulation, suggesting that the induction of autophagy was coupled with apoptosis. Therefore, our results indicate that GW8510 triggered the death of CRC cells through inducing apoptosis and autophagy.

To confirm the role of RRM2 in GW8510-induced autophagy, HCT116 cells were transfected with a RRM2 siRNA or a RRM2-overexpressing plasmid, and then subjected to western blot analysis. As shown in Figure 3d, knockdown of RRM2 can promote autophagy as indicated by LC3-II accumulation and p62 downregulation. In addition, GW8510-induced autophagy was enhanced when RRM2 was knocked down. However, overexpression of RRM2 did not alter GW8510-induced autophagy (Figure 3e). These results suggest that GW8510 induces autophagy in both RRM2-dpendent and RRM2-independent manners.

### GW8510 exhibits anti-CRC effect in a p53-independent manner

**Figure 4:**
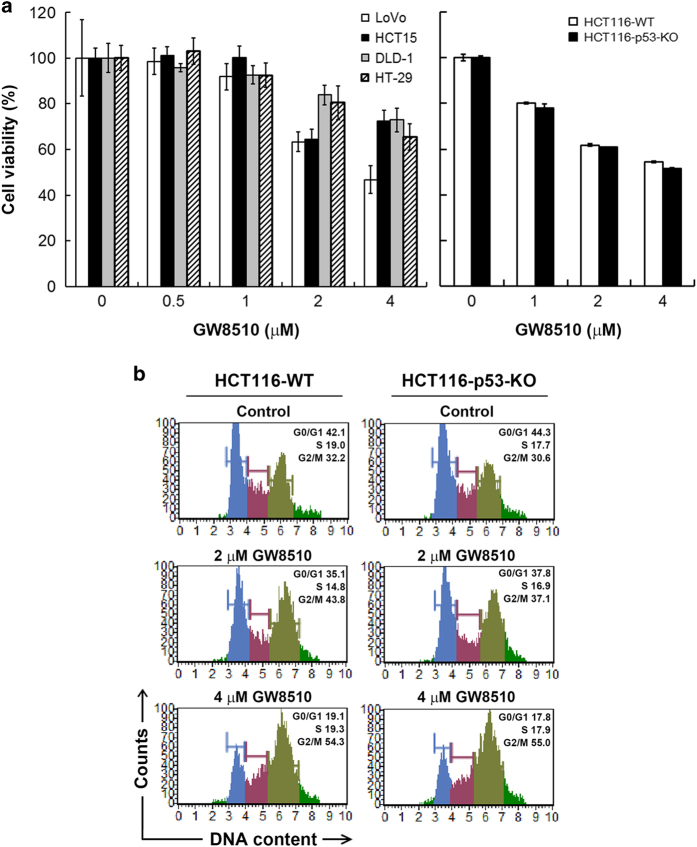
GW8510 induced anti-CRC activity regardless of p53 status. (**a**) LoVo, HCT15, DLD-1, HT-29, HCT116-wild-type (WT), and HCT116 p53-knockout (KO) cells were treated with various doses of GW8510 for 72 h. The cell viability was determined by an MTT assay. (**b**) HCT116 cells were treated the different doses of GW8510 for 24 h, and the cell cycle was analyzed by flow cytometry as described in ‘Materials and Methods’.

The anticancer effect of GW8510 on other human CRC cell lines was further investigated. As shown in Figure 4a (left part), GW8510 exhibited potent anticancer activity against LoVo, HCT15, DLD-1, and HT-29 cells. The results also indicated that p53 mutant cells (HCT15, DLD-1, and HT-29) were more resistant to GW8510 than p53 wild-type cells (LoVo and HCT116) (Figure 4a). To investigate the role of p53, isogenic p53-knockout HCT116 cells were used. However, p53 deficiency did not alter the effect of GW8510 on cell viability (Figure 4a, right part). Consistently, cell cycle analysis showed that GW8510 induced G2/M cell cycle arrest in both p53 wild-type and knockout HCT116 cells (Figure 4b). Therefore, these results suggest that GW8510 was an effective anti-CRC agent regardless of p53 status.

### Impaired autophagic flux is associated with the resistance of CRC cells to GW8510

**Figure 5:**
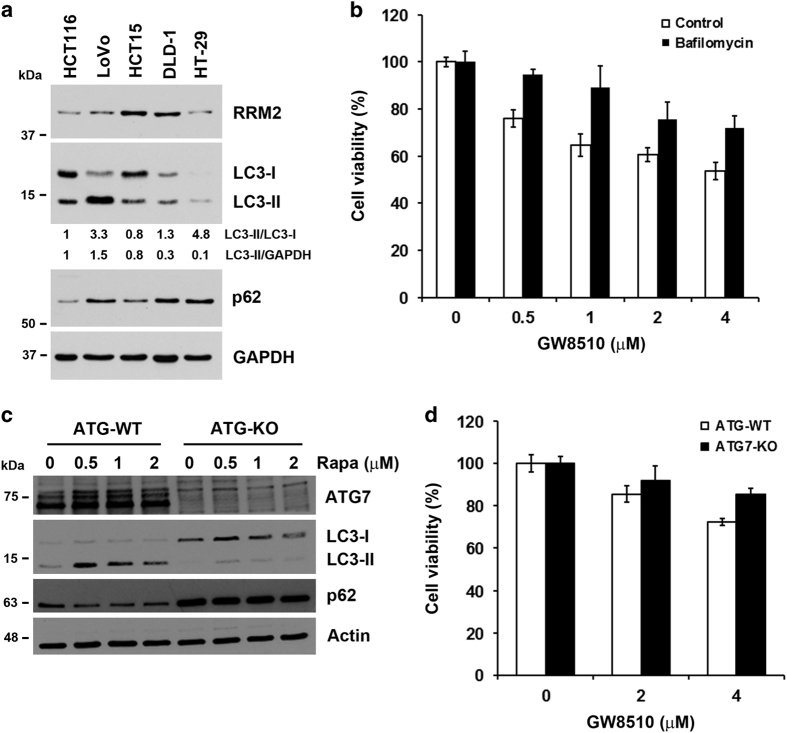
Autophagy deficiency was associated with the sensitivity of CRC cells to GW8510. (**a**) The protein expressions in HCT116, LoVo, HCT15, DLD-1, and HT-29 cells were analyzed by western blots. The ratio of LC3-II to LC3-I or GAPDH was quantified. (**b**) HCT116 cells were treated the different doses of GW8510 for 72 h in the absence or presence of 10 nM bafilomycin. The cell viability was determined by an MTT assay. (**c**) HCT116 cells were treated with different doses of rapamycin (Rapa) for 24 h. The protein expressions were analyzed by western blots. (**d**) ATG7-wild-type (ATG7-WT) and ATG7-knockout (ATG7-KO) DLD-1 cells were treated with different doses of GW8510 for 72 h. The cell viability was determined by an MTT assay.

To further investigate the mechanism responsible for the differential sensitivities of CRC cells to GW8510, the relative RRM2 expression levels in these cells were compared by western blot analysis. As shown in Figure 5a, both HCT15 and DLD-1 showed the highest level of RRM2. However, overexpression of RRM2 could not fully explain their differential sensitivities to GW8510 because of the relatively low RRM2 expression in HT-29 cells (Figure 5a). As GW8510 induced autophagic cell death of CRC cells, we hypothesized that, in addition to the level of RRM2, the endogenous abilities of CRC cells to undergo autophagy also determined the cell fate. Thus, the expression of LC3 and p62 was examined by western blot analysis to estimate the overall autophagic flux in cells.^19^ As shown in Figure 5a, GW8510-sensitive cells (HCT116 and LoVo) had higher LC3-II accumulation and lower p62 expression than GW8510-resistant cells (HCT15, DLD-1, and HT-29). Although p62 expression in LoVo cells were relatively high among these cells, the accelerated autophagic flux (higher LC3-II/LC3-I ratio) might be able to compensate for high p62 expression. To confirm the role of autophagy in the sensitivity of GW8510, HCT116 cells were treated with bafilomycin A1 to block autophagy flux. Bafilomycin A1 is a vacuolar-type H^+^-ATPase inhibitor that blocks autophagosome-lysosome fusion.^19^ Treatment with bafilomycin attenuated the anticancer activity of GW8510 (Figure 5b). Furthermore, autophagy-deficient ATG7-knockout (ATG7-KO) DLD-1 cells were used. The impairment of autophagy in ATG7-KO cells was examined by treating with an autophagy inducer, rapamycin. As shown in Figure 5c, rapamycin could trigger autophagy (LC3-II accumulation and p62 degradation) in ATG7-wild-type (ATG7-WT) DLD-1 cells, but not in ATG7-KO cells. In addition, p62 accumulation was found in ATG7-KO cells (Figure 5c). Consistent with the effect by bafilomycin (Figure 5b), ATG7-KO cells were resistant to GW8510 treatment (Figure 5d). Taken together, both RRM2 overexpression and impaired autophagic flux in CRC cells contributes the resistance of GW8510.

## Discussion

Their roles of three RR subunits in tumorigenesis are quite different. RRM2 increases tumorigenic potential via cooperating with a variety of oncogenes, whereas RRM1 has malignancy-suppressing activity.^20,21^ Metastasis-suppressing potential of RRM2B is found in human cancer patients.^22^ Moreover, RRM2 is an independent prognostic factor and predicts poor survival of CRC. It is also a potential predictor for identifying good responders to chemotherapy for CRC.^23^ In contrast to RRM2, both RRM1 and RRM2B are associated with a better prognosis for cancer patients.^22,24–27^ Therefore, it is suggested that, besides the antiproliferative activity of RR inhibition, the specific inhibition of RRM2 expression might provide additional anticancer benefits.^28^ Given the fact that current clinically used RR inhibitors, such as gemcitabine and hydroxyurea, lack specific activity for RRM2, the development of novel RRM2 inhibitors is of immediate importance. In this study, we showed that GW8510 specifically inhibited RRM2 protein expression without the alteration of RRM1 expression. GW8510 might be an attractive compound for developing novel specific RRM2 inhibitors.

GW8510 is found to be one of the candidate compounds to treat CRC by a recent study using the Functional Module Connectivity Map (FMCM) to search repositioned drugs.^29^ FMCM is a CMAP-based method for the discovery of ontology-specific repositioning drugs. It uses multiple functional gene modules constructed from gene expression data of normal and colorectal adenoma patients to query the CMAP.^29^ The beneficial effects of GW8510 include functional modules of cell proliferation, signal transduction, DNA replication, apoptosis, cell cycle, transcription, and RNA metabolic process.^29^ This study and ours demonstrate the potential application of GW8510 for the treatment of CRC. In addition, our study suggests that these processes altered by GW8510 might serve as end points of RRM2 inhibition.

A recent study reveals a reciprocal regulation of autophagy and dNTP pools in human cancer cells through modulation of RR activity or RRM2 expression.^18^ It is found that starvation- or rapamycin-induced autophagy is accompanied by a decrease in RR activity and dNTP pools in human cancer cells. In addition, downregulation of RRM2 or treatment with RR inhibitor, hydroxyurea, induces autophagy.^18^ Accordingly, synergistic anticancer effect of autophagy induction and RRM2 inhibition was further demonstrated.^30^ In this study, we also found that knockdown of RRM2 by siRNA was able to induce autophagy and enhance GW8510-induced autophagy, suggesting the promoting role of RRM2 inhibition. However, overexpression of RRM2 was not sufficient to rescue GW8510-induced autophagy. Possibly, GW8510 could induce autophagy through other pathways independent of RRM2 inhibition. Moreover, we also found that impaired autophagic flux might result in the resistance of cancer cells to GW8510. Thus, combination therapy with GW8510 and autophagy inducer, such as rapamycin, could augment the therapeutic effect.

In conclusion, we report that RRM2 is a potential molecular target for treating human CRC. CMAP, a gene expression signature-based approach, could be an attractive strategy for drug repositioning to discover novel RRM2 inhibitors. Our result indicates that GW8510, originally developed as a cyclin-dependent kinase inhibitor, could inhibit RRM2 expression and autophagic cell death in human CRC cells.

## Materials and Methods

### Materials

RPMI-1640 medium, L-glutamine, sodium pyruvate, and Antibiotic-Antimycotic Solution (penicillin G, streptomycin, and amphotericin B), and Lipofectamine RNAiMAX Transfection Reagent were purchased from Life Technologies (Gaithersburg, MD, USA). Fetal bovine serum was purchased from Gibco (Grand Island, NY, USA). RRM1, RRM2, p62, LC3B, Actin, and GAPDH antibodies were purchased from GeneTex (Hsinchu, Taiwan). The PARP1 antibody was purchased from Cell Signaling Technology (Beverly, MA, USA). The ATG7 antibody was purchased from Santa Cruz (Island, CA, USA). Horseradish peroxidase-labeled goat anti-rabbit and anti-mouse secondary antibodies were purchased from Jackson ImmunoResearch (West Grove, PA, USA). pcDNA3-RRM2 plasmid was purchased from Addgene (Cambridge, MA, USA). PolyJet *In Vitro* DNA Tranfection Reagent was purchased from SignaGen Laboratories (Ijamsville, MD, USA). siGENOME human RRM2 SMARTpool siRNAs and siGENOME Non-Targeting human siRNA Pool were purchased from Dharmacon (Lafayette, CO, USA). Rapamycin and phenoxybenzamine were purchased from Cayman Chemical (Ann Arbor, MI, USA). Doxorubicin and bafilomycin A1 were purchased from LC Laboratories (Woburn, MA, USA). Daunorubicin was purchased from Biovision (Mountain View, CA, USA). ZVAD-FMK was purchased from ApexBio Technology (Houston, TX, USA). GW8510, MG132, 3-(4,5-Dimethylthiazol-2-yl)-2,5-diphenyl tetrazolium bromide (MTT), dimethyl sulfoxide (DMSO), propidium iodide (PI), and ribonuclease A (RNase A) were purchased from Sigma (St. Louis, MO, USA). Protease and phosphatase inhibitor cocktails were purchased from Roche (Indianapolis, IN, USA). Other chemicals or reagents not specified were purchased from OneStar Biotechnology (New Taipei City, Taiwan).

### Cell culture

Human colon cancer cells (HCT116, LoVo, RKO, HCT15, DLD-1, and HT-29) were kindly provided by Prof. Ya-Wen Cheng (Taipei Medical University, Taipei, Taiwan). ATG7-wildtype (ATG7-WT) and ATG7-knockout (ATG7-KO) DLD-1 cells were purchased from Horizon Discovery (Cambridge, UK). These cells were cultured in RPMI-1640 medium supplemented with 10% fetal bovine serum, 1 mM sodium pyruvate, 1% L-glutamine, and 1% Antibiotic-Antimycotic Solution, and incubated at 37 °C in a humidified incubator containing 5% CO_2_.

### Cell viability assay

Cell viability was measured with an MTT assay. Cells were plated in 96-well plates and treated with drugs. After 72 h of incubation, 0.5 mg/ml of MTT was added to each well for an additional 4 h. The blue MTT formazan precipitate was then dissolved in 200 *μ*l of DMSO. The absorbance at 550 nm was measured on a multiwell plate reader.

### Western blot analysis

Cells were lysed in an ice-cold buffer containing 50 mM Tris-HCl (pH 7.5), 150 mM NaCl, 1 mM MgCl_2_, 2 mM EDTA, 1% NP-40, 10% glycerol, 1 mM DTT, 1× protease inhibitor cocktail, and 1× phosphatase inhibitor cocktail at 4 °C for 30 min. Cell lysates were separated on a sodium dodecyl sulfate-polyacrylamide gel, and then transferred electrophoretically onto a Hybond-C Extra nitrocellulose membrane (GE Healthcare, Piscataway, NJ, USA). The membrane was pre-hybridized in 20 mM Tris-HCl (pH 7.5), 150 mM NaCl, 0.05% Tween-20 (TBST buffer), and 5% skim milk for 1 h, and then transferred to a solution containing 1% bovine serum albumin/TBST and a primary antibody and incubated overnight at 4 °C. After washing with the TBST buffer, the membrane was submerged in 1% bovine serum albumin/TBST containing an horseradish peroxidase-conjugated secondary antibody for 1 h. The membrane was washed with TBST buffer, and then developed with an enhanced chemiluminescence system (Perkin-Elmer, Boston, MA, USA) and exposed to x-ray film (Roche).

### Flow cytometric analyses of the cell cycle

Cells were plated in 6-well plates for 24 h, and then treated with complete medium containing drugs for 24 h. Floating and adherent cells were harvested, immediately fixed with 75% ethanol, and stored at −20 °C. Cells were stained in staining buffer (10 *μ*g/ml PI and 100 *μ*g/ml RNase A) for 30 min and then analyzed on a Muse Cell Analyzer (Merck Millipore, Billerica, MA, USA).

### Transient transfection

For RRM2 overexpression, human RRM2-overexpressing (pcDNA3-RRM2) and its control (pcDNA3) plasmids were transiently transfected into cells with PolyJet *In Vitro* DNA Tranfection Reagent according to the manufacturer’s instructions. For siRNA knockdown analysis, human RRM2 and control siRNAs were transiently transfected into cells with Lipofectamine RNAiMAX Transfection Reagent according to the manufacturer’s instructions. Twenty-four hours after transfection, the transfection mixture was replaced with fresh complete medium and cells were used for further experiments.

## Figures and Tables

**Table 1:** The gene expression signatures of compounds most positively (mean score>0.7 and *P*<0.01) correlated with that of RRM2 siRNA

*CMAP drug*	*Function*	*Mean score*	*No of instances*	*Enrichment*	*P value*	*Specificity*	*Percent non-null*
Phenoxybenzamine	Non-selective, irreversible alpha antagonist	0.892	4	0.986	0	0.0446	100
Doxorubicin	Topoisomerase II inhibitor	0.818	3	0.958	0.0001	0.0447	100
5248896	Unknown	0.812	2	0.974	0.00109	0	100
GW8510	CDK inhibitor	0.754	4	0.915	0.00004	0.0884	100
Trioxysalen	A furanocoumarin and a psoralen derivative	0.748	4	0.765	0.00581	0.0261	100
0175029-0000	CDK inhibitor	0.733	6	0.814	0.00008	0.0353	100
Daunorubicin	Topoisomerase II inhibitor	0.728	4	0.883	0.0002	0.0404	100
Thioguanosine	A purine analog showing antineoplastic activity	0.713	4	0.823	0.00167	0.0412	100
Ellipticine	Topoisomerase II inhibitor	0.711	4	0.844	0.00097	0.0608	100
Sulconazole	Antifungal medication of the imidazole class	0.708	4	0.738	0.00923	0.0375	100
Morantel	Anthelmintic for veterinary use	0.701	5	0.822	0.00038	0	100

Abbreviation: CDK, cyclin-dependent kinase. The results were ranked by their mean scores.
