# Supplementary material for: Repositioning of a cyclin-dependent kinase inhibitor GW8510 as a ribonucleotide reductase M2 inhibitor to treat human colorectal cancer
Source: Cell Death Discov. 2016 May 9;2:16027–. doi: 10.1038/cddiscovery.2016.27 (PMC4979501; doi:10.1038/cddiscovery.2016.27)
Supplement: Supplementary Information [file cddiscovery201627-s1.pdf]

Supplementary Information

Repositioning of a cyclin-dependent kinase inhibitor GW8510 as a ribonucleotide reductase M2 inhibitor to treat human colorectal cancer

Yao-Yu Hsieh[1,2], Chia-Jung Chou[1,3], Hsiang-Ling Lo[1,3], and Pei-Ming Yang[1,3,*]

[1]PhD Program for Cancer Biology and Drug Discovery, College of Medical Science and Technology, Taipei Medical University and Academia Sinica, Taipei, Taiwan

[2]Division of Hematology and Oncology, Shuang Ho Hospital, Taipei Meidcal University, Taipei, Taiwan

[3]Graduate Institute of Cancer Biology and Drug Discovery, College of Medical Science and Technology, Taipei Medical University, Taipei, Taiwan

***To whom correspondence should be addressed:**

Pei-Ming Yang, Graduate Institute of Cancer Biology and Drug Discovery, College of Medical Science and Technology, Taipei Medical University; 250 Wu-Hsing Street, Taipei 11031, Taiwan; Phone: 886-2-27361661 ext. 7629; Fax: 886-2-26558562; E-mail: yangpm@tmu.edu.tw

Supplementary Information includes:

Supplementary Figures S1 and S2

Supplementary Table S1

Legends to Supplementary Figures

Fig. S1. Effect of doxorubicin and daunorubicin on cell viability and RRM2 expression in HCT116 cells. (A) The chemical structures of phenoxybenzamine (PBZ). **(B)** HCT116 and DLD-1 cells were treated with various doses of phenoxybenzamine for 72 h. The cell viability was determined by an MTT assay. **(C)** HCT116 cells were treated with various doses of phenoxybenzamine for 24 h. The protein expressions were analyzed by Western blots.

Fig. S2. Effect of doxorubicin and daunorubicin on cell viability and RRM2 expression in HCT116 cells. (A) The chemical structures of doxorubicin (Doxo) and daunorubicin (Dauno). **(B)** HCT116 cells were treated with various doses of doxorubicin and daunorubicin for 72 h. The cell viability was determined by an MTT assay. **(C)** HCT116 cells were treated with various doses of doxorubicin and daunorubicin for 24 h. The protein expressions were analyzed by Western blots.

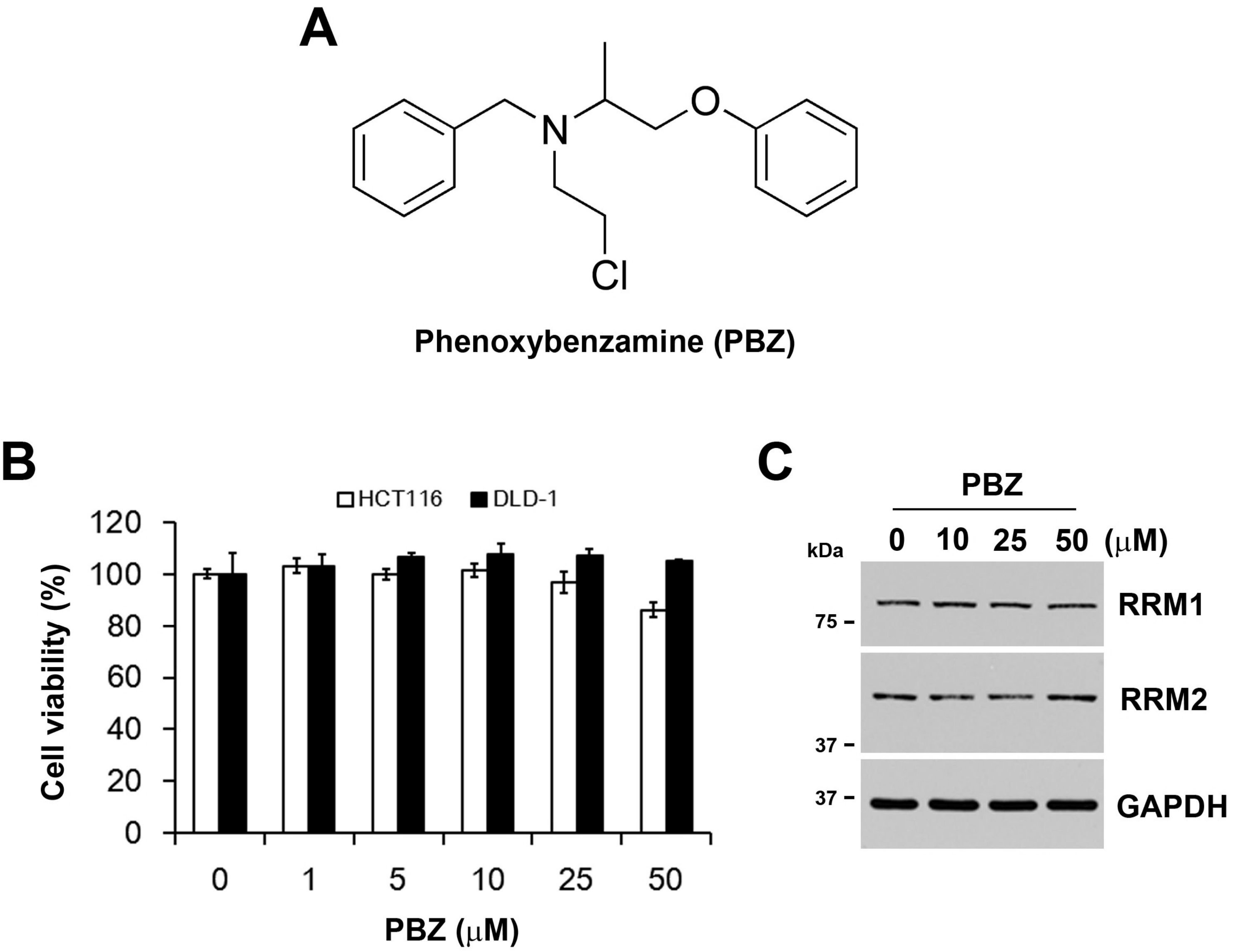
A
O
N
Cl
Phenoxybenzamine (PBZ)
B
HCT116
DLD-1
Cell viability (%)
120
100
80
60
40
20
0
0
1
5
10
25
50
PBZ (μM)
C
PBZ
0
10
25
50
(μM)
kDa
75
37
37
RRM1
RRM2
GAPDH

Suppl. Fig. S1

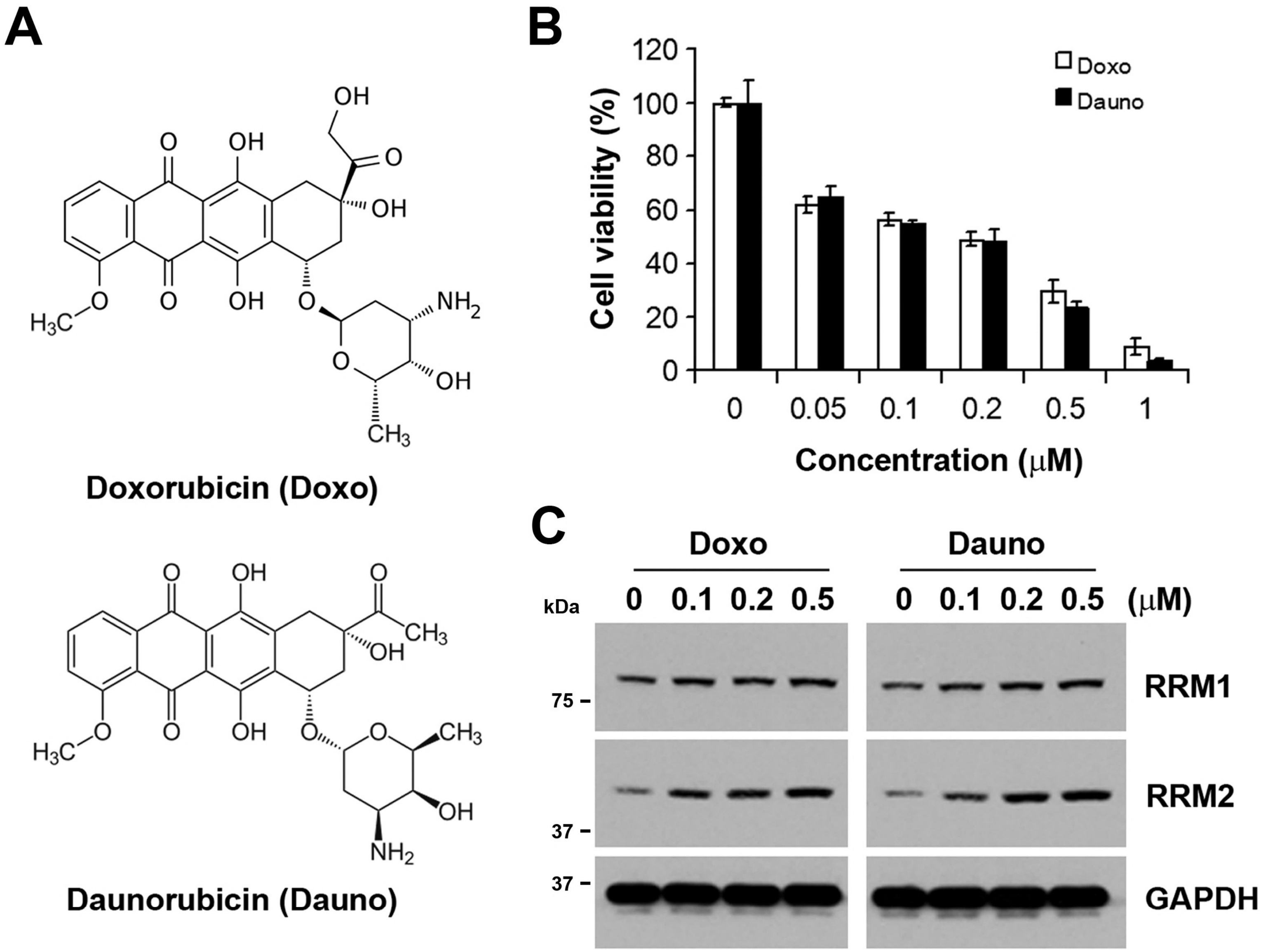
A
Doxorubicin (Doxo)
Daunorubicin (Dauno)
B
Cell viability (%)
120
100
80
60
40
20
0
Doxo
Dauno
0
0.05
0.1
0.2
0.5
1
Concentration (μM)
C
Doxo
Dauno
kDa
0 0.1 0.2 0.5
0 0.1 0.2 0.5 (μM)
75
37
37
RRM1
RRM2
GAPDH

Suppl. Fig. S2

Supplementary Table S1. Differentially expressed genes induced in RRM2 siRNA-transfected SW480 cells. This dataset (GSE15212) was obtained from NCBI GEO.

Gene symbol	Gene title	Fold change (Log2)
RFTN1	raftlin, lipid raft linker 1	1.82
ANGPTL4	angiopoietin-like 4	1.62
SAMD9	sterile alpha motif domain containing 9	1.51
CHI3L1	chitinase 3-like 1 (cartilage glycoprotein-39)	1.44
FGFR3	fibroblast growth factor receptor 3	1.38
C19orf57	chromosome 19 open reading frame 57	1.32
PAG1	phosphoprotein associated with glycosphingolipid microdomains 1	1.29
AZGP1	alpha-2-glycoprotein 1, zinc-binding	1.28
STOM	stomatin	1.27
TECPR2	tectonin beta-propeller repeat containing 2	1.25
PLEK2	pleckstrin 2	1.20
SLPI	secretory leukocyte peptidase inhibitor	1.20
SELENBP1	selenium binding protein 1	1.19
ZNF367	zinc finger protein 367	1.19
IL11	interleukin 11	1.17
TBX2	T-box 2	1.17
NOV	nephroblastoma overexpressed	1.16
RASGEF1A	RasGEF domain family, member 1A	1.15
KCNQ2	potassium voltage-gated channel, KQT-like subfamily, member 2	1.15
GPER	G protein-coupled estrogen receptor 1	1.15
COCH	cochlin	1.14
CH25H	cholesterol 25-hydroxylase	1.12
SUSD3	sushi domain containing 3	1.11
ABCB1	ATP-binding cassette, sub-family B (MDR/TAP), member 1	1.11
KRTAP2-3	keratin associated protein 2-3	1.10
APOC1	apolipoprotein C-I	1.10
TLR2	toll-like receptor 2	1.10
G0S2	G0/G1switch 2	1.10
TRIM15	tripartite motif containing 15	1.09
LOC100506737	uncharacterized LOC100506737	1.09

PRRG4	proline rich Gla (G-carboxyglutamic acid) 4 (transmembrane)	1.08
LAT2	linker for activation of T cells family, member 2	1.08
TMEM173	transmembrane protein 173	1.08
RAC2	ras-related C3 botulinum toxin substrate 2 (rho family, small GTP binding protein Rac2)	1.07
TFF3	trefoil factor 3 (intestinal)	1.06
CLDN1	claudin 1	1.06
STYK1	serine/threonine/tyrosine kinase 1	1.05
CYP3A5	cytochrome P450, family 3, subfamily A, polypeptide 5	1.05
CALCA	calcitonin-related polypeptide alpha	1.03
APOLD1	apolipoprotein L domain containing 1	1.03
PROCR	protein C receptor, endothelial	1.03
NOS2	nitric oxide synthase 2, inducible	1.03
ARRDC4	arrestin domain containing 4	1.02
CCL26	chemokine (C-C motif) ligand 26	1.01
SH3YL1	SH3 domain containing, Ysc84-like 1 (S. cerevisiae)	1.01
CD82	CD82 molecule	1.00
AHNAK	AHNAK nucleoprotein	-1.01
OMA1	OMA1 zinc metallopeptidase	-1.06
RCSD1	RCSD domain containing 1	-1.16
H1F0	H1 histone family, member 0	-1.16
RRM2	ribonucleotide reductase M2	-2.54
